# Editorial: Adverse Health Consequences of Excessive Smartphone Usage

**DOI:** 10.3389/fpubh.2021.689968

**Published:** 2021-07-26

**Authors:** Paul H. Lee

**Affiliations:** Department of Health Sciences, University of Leicester, Leicester, United Kingdom

**Keywords:** smartphone, mobile, screen, technology, addiction

The use of smartphones has been increasing rapidly in recent years. Following their growing dominance in our lives, there are numerous research investigations about the impact of smartphone usage on health outcomes. In this Research Topic, we have collected four contributions about the adverse health consequences of excessive smartphone usage.

Short video sharing has becoming popular for smartphone users, and TikTok, which was originated in China, has become one of the most popular apps in the world since its foundation in 2016. Montag et al. reviewed the psychological impacts of TikTok on its users. They found that (1) the reason of people use TikTok could be derived using gratification theory, social impact theory, and self-determination theory; (2) most TikTok users are young and motivated, and (3) the usage pattern of TikTok is yet to be studied, but shown to be very different of the usage patterns of other social media platforms such as Instagram or Facebook.

Smartphone addiction is an emerging risk factors of mental health problems, and its identification was of utmost importance. Park et al. identified significant characteristics of smartphone addicted users among 600 Korean adults using online survey. Using the validated Korean Smartphone Addiction Proneness Scale for Adults as a screening tool, it was identified that 17% of them were smartphone addicted. They showing that they had more weekend average usage time, habitual smartphone behavior, addictive smartphone behavior, social usage, and process usage than non-addicted users. The authors had developed a smartphone addiction management app to help addicted users to manage their smartphone usage. Along this research direction, Peterka-Bonetta et al. examined the association between personality and smartphone addiction from 773 young adults in 59 countries. Results revealed that those neurotic, unconscientious, not agreeable, social anxious, and impulsive, were more likely to be addicted with smartphone.

Recently, mobile games has increased in popularity and replacing computer and console games, and mobile game addiction has several unique features against other types of game addiction. Wang et al. surveyed 600 Chinese junior high school students and found that mobile game addicted adolescents had elevated social anxiety, depression, and loneliness.

While all these studies contributed to our knowledge on excessive smartphone usage, further research in this domain is warranted. Most existing studies examining the impact of excessive smartphone usage adopted a cross-sectional approach so that reverse causation could not be eliminated, and multiple time point studies are required to establish the causal direction. In addition, majority of the smartphone addiction measures relied on self-reports, and objective measurements, such as monitoring of smartphone activity, should be further investigated.

## Author Contributions

The author confirms being the sole contributor of this work and has approved it for publication.

## Conflict of Interest

The author declares that the research was conducted in the absence of any commercial or financial relationships that could be construed as a potential conflict of interest.

## Publisher's Note

All claims expressed in this article are solely those of the authors and do not necessarily represent those of their affiliated organizations, or those of the publisher, the editors and the reviewers. Any product that may be evaluated in this article, or claim that may be made by its manufacturer, is not guaranteed or endorsed by the publisher.

